# Sex-Specific Longitudinal Changes in Metabolic, Endocrine, Renal, Cardiovascular, and Inflammatory Biomarkers of Vaccinated COVID-19 Survivors: 30-Month Follow-Up Study

**DOI:** 10.3390/medicina61091510

**Published:** 2025-08-22

**Authors:** Ceren Gur, Sezen Kumas Solak, Erdal Gundogan, Fatih Pektas, Hafize Uzun

**Affiliations:** 1 Department of Internal Medicine, Bağcılar Training and Research Hospital, University of Health Sciences, 34100 Istanbul, Türkiye; 2 Department of Anesthesiology and Reanimation, Bağcılar Training and Research Hospital, University of Health Sciences, 34100 Istanbul, Türkiye; sezenkumassolak@gmail.com; 3 Department of Internal Medicine, Faculty of Medicine, Istanbul Atlas University, 34403 Istanbul, Türkiye; erdal.gundogan@atlas.edu.tr (E.G.); fatif.pektas@atlas.edu.tr (F.P.); 4 Department of Medical Biochemistry, Faculty of Medicine, Istanbul Atlas University, 34403 Istanbul, Türkiye; huzun59@hotmail.com

**Keywords:** COVID-19, sex differences, inflammation, metabolism, thyroid function, vaccination

## Abstract

*Background and Objectives*: Sex-based disparities in COVID-19 outcomes are well-documented, with men experiencing greater acute severity and women showing increased vulnerability to post-viral syndromes. However, longitudinal immunometabolic trajectories in vaccinated individuals remain underexplored. In this study, sex-based differences in long-term metabolic, endocrine, renal, cardiovascular, and inflammatory responses were investigated among vaccinated individuals recovering from SARS-CoV-2 infection. *Materials and Methods*: This retrospective single-center cohort study included 426 adults (199 females, 227 males) with PCR-confirmed symptomatic COVID-19 and at least two vaccine doses. Serial assessments were conducted at baseline, 18-, 24-, and 30-month post-infection. Parameters included fasting glucose, HbA1c, lipid profile, thyroid function, renal markers, CRP, D-dimer, fibrinogen, troponin, and hematologic indices. Statistical analyses assessed longitudinal changes and sex-stratified correlations. *Results*: Fasting glucose and HbA1c levels significantly declined over time, more prominently in males. Glucose correlated with age and BMI only in females. Lipid levels remained largely unchanged, although males had higher baseline triglycerides. Females showed rising TSH levels and persistently lower free T3; males exhibited higher creatinine, urea, and troponin levels throughout. Inflammatory markers declined significantly in both sexes, with males displaying higher CRP and troponin, and females showing sustained fibrinogen elevation and a temporary lymphocyte surge. D-dimer was elevated in females at the 30-month point. *Conclusions*: Sex-specific physiological recovery patterns were evident among vaccinated COVID-19 survivors. Males exhibited earlier metabolic and cardiac alterations, while females had more persistent endocrine and inflammatory shifts. These findings underscore the need for sex-tailored long-term monitoring strategies prioritizing early metabolic and cardiac screening in men and prolonged immunoendocrine surveillance in women.

## 1. Introduction

The COVID-19 pandemic, caused by SARS-CoV-2, has prompted extensive investigation into its long-term effects, particularly in vaccinated populations. Although vaccination reduces acute disease severity, emerging evidence suggests persistent alterations in metabolic, endocrine, and inflammatory pathways [1,2,3]. These changes may impact long-term health and quality of life. Biological sex is a key determinant of immune responses, influencing susceptibility, disease course, and recovery following SARS-CoV-2 infection and vaccination [4,5].

Research indicates that men tend to experience more severe outcomes from COVID-19, with higher rates of hospitalization, thromboinflammatory complications, and mortality [1,6,7,8]. These outcomes are often attributed to sex-based differences in immunological responses, including elevated levels of pro-inflammatory cytokines, impaired T cell responses, and variations in the expression of ACE2 and TMPRSS2 receptors. Conversely, women generally exhibit stronger innate and adaptive immune responses but paradoxically are more susceptible to post-viral syndromes, autoimmune sequelae, and adverse reactions related to vaccines or infections [5,9]. The long-term effects of SARS-CoV-2, known as long COVID complications, which are more common in women than men, are also linked to depression, reduced physical activity, and poor lifestyle habits [10]. Notably, long COVID frequently involves cardiometabolic and endocrine abnormalities, especially among women. This underscores the importance of considering sex-specific risk stratification [11]. Additionally, SARS-CoV-2-mediated damage and/or vaccination may influence post-infection immunometabolism, affecting inflammation, glucose levels, lipid metabolism, renal function, and thyroid regulation in the post-pandemic era [12,13,14,15].

With widespread COVID-19 vaccination, most individuals now experience infection post-vaccination, which may alter disease progression and recovery. However, data on long-term outcomes in vaccinated populations remain limited. This study focuses on vaccinated patients to better understand sex-specific metabolic and inflammatory changes after COVID-19, providing relevant insights for current clinical management and follow-up. This retrospective cohort study explores sex-based differences in metabolic, endocrine, and inflammatory responses to SARS-CoV-2 infection in vaccinated individuals. Serial blood samples were collected over 30 months. Baseline and follow-up assessments included markers of glucose metabolism, thyroid function, inflammation, coagulation, and cardiac status. The study aims to clarify the role of biological sex in post-infection immunometabolic dynamics to support personalized recovery strategies.

## 2. Materials and Methods

### 2.1. Study Design and Setting

This study was designed as a retrospective, single-center cohort investigation to evaluate the long-term alterations in metabolic, endocrine, renal, cardiovascular, and inflammatory parameters in adults recovering from COVID-19 confirmed via polymerase chain reaction (PCR). All participants had received at least two doses of SARS-CoV-2 vaccines. The study took place at the COVID-19 Ward of Bağcılar Training and Research Hospital, University of Health Sciences in Istanbul, Türkiye. Data collection took place at four intervals over 30 months, at the baseline (T_0_), 18 months (T_1_), 24 months (T_2_), and 30 months (T_3_).

### 2.2. Study Population and Cohort Characteristics

The eligibility criteria included adults aged 18 or over with confirmed symptomatic SARS-CoV-2 infection, as determined by reverse transcription polymerase chain reaction (RT-PCR), who had received at least two doses of a SARS-CoV-2 vaccine (either mRNA, BNT162b2 [Comirnaty], or inactivated, CoronaVac) prior to infection. Any additional vaccine doses administered during the 30-month follow-up were recorded, and the total number of doses for each participant is indicated in Table 1. Vaccination status, including type and number of doses, was verified through institutional medical records and the Turkish National Health Information System. Patients with incomplete vaccination documentation, immunocompromised status, or severe comorbidities likely to confound biomarker profiles were excluded. Additionally, the exclusion criteria included individuals who were pregnant or lactating, those with malignancies, those with cognitive dysfunction, those who did not provide consent to participate in the study, and those who were objectively unable to participate due to physical or mental limitations. Based on PCR-confirmed results in the medical records, no participants were reported to have had confirmed reinfection either before recruitment or during the 30-month follow-up. Therefore, subgroup analyses related to reinfection were not feasible and are noted as a limitation.

Participants’ symptoms at the time of hospitalization related to SARS-CoV-2 infection met WHO criteria [16]. All included participants had moderate COVID-19, requiring hospitalization in the COVID-19 ward; none were admitted to the intensive care unit (ICU) or followed as outpatients. All individuals were treated at the COVID-19 Ward from January 2020 to December 2022. The final cohort comprised 426 vaccinated individuals, stratified by sex into female (n = 199) and male (n = 227) subgroups. The specific vaccine type received (mRNA BioNTech or inactivated SINOVAC) is also reported in the cohort characteristics. A priori sample size estimation was conducted using G*Power 3.1.9, assuming a medium effect size (f = 0.25), α = 0.05, and yielded a statistical power (1−-β) of 0.993, confirming adequacy for detecting between-group differences.

Pulmonary outcomes were not included because the study cohort consisted primarily of patients with mild to moderate COVID-19 who did not require intensive respiratory interventions, and longitudinal pulmonary function assessments were not consistently available across all follow-up intervals. The study, therefore, focused on systemic biomarkers.

### 2.3. Data Acquisition and Laboratory Assessments

Demographic parameters, including age, sex, and body mass index (BMI), were retrieved from electronic health records. BMI was calculated using the standard formula: weight (kg) divided by height squared (m^2^). Blood pressure measurements were obtained using automated sphygmomanometers under clinical conditions.

Venous blood samples were collected at each time point and processed according to standard clinical laboratory protocols. Biochemical and endocrine parameters—including fasting glucose, hemoglobin A1c (HbA1c), serum urea, creatinine, thyroid-stimulating hormone (TSH), free triiodothyronine (T3), free thyroxine (T4), total cholesterol, triglycerides, and low-density lipoprotein (LDL) cholesterol were measured using the Roche-Hitachi Cobas 8000 autoanalyzer (Roche Diagnostics, Mannheim, Germany). Inflammatory biomarkers, including C-reactive protein (CRP), fibrinogen, and D-dimer, were quantified using immunoturbidimetric assays on the Roche Cobas 8000 analyzer, following the manufacturer’s protocols. In these assays, antigen-antibody complexes form in proportion to the analyte concentration, and turbidity is measured photometrically. High-sensitivity cardiac troponin (hs-cTn) was measured using a chemiluminescence immunoassay (CLIA) on the same platform. In this method, target antigens bind to specific antibodies labeled with a chemiluminescent substrate; the emitted light is directly proportional to the concentration of the analyte.

Hematological assessments, including total white blood cell (WBC) counts and differential counts for neutrophils (NEU) and lymphocytes (LY), were analyzed using the Mindray- BS-2000m (Mindray, Shenzhen, China) hematology analyzer on EDTA-treated whole blood samples. Additionally, participants’ blood pressure measurements were documented.

### 2.4. Ethical Considerations

The study protocol was reviewed and approved by the Institutional Ethics Committee of Bağcılar Training and Research Hospital (Approval Number: 2025/04/06/038; Approval Date: by 11 April 2025, and was conducted in accordance with the principles of the Declaration of Helsinki and local regulatory requirements. Given the retrospective nature of the study, informed consent was not required, and all patient data were anonymized. Consent was obtained using the “opt-out method”, thereby allowing patients to decline participation.

### 2.5. Statistical Analysis

Statistical analyses were carried out using GraphPad Prism version 10.5 (GraphPad Software, La Jolla, CA, USA). Continuous variables were presented as mean ± standard error of the mean (SEM) or median with interquartile range, depending on data distribution, while categorical variables were expressed as frequencies and percentages. The Shapiro–Wilk test was employed to assess the normality of continuous data. Fisher’s exact test was used to evaluate associations between categorical variables. A Kruskal–Wallis analysis with Dunn’s post hoc test was performed to evaluate multiple comparisons. The Mann–Whitney U test was used to analyze differences between the two groups. Correlations between continuous parameters were analyzed using Spearman’s correlation, with correlation strengths interpreted as weak (0.20–0.39), moderate (0.40–0.59), strong (0.60–0.79), or very strong (0.80–1.00). Negative (−) r values indicate negative correlations, while positive (+) r values indicate positive correlations. A *p*-value < 0.05 was considered statistically significant for all tests. *p*-values shown in tables reflect inter-sex comparisons at each time point and overall.

## 3. Results

**Figure 1:**
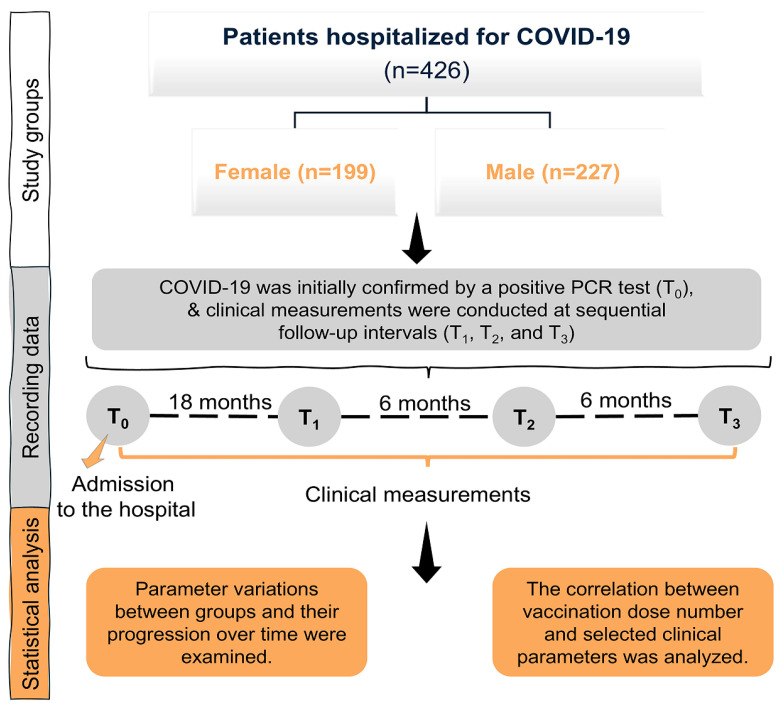
Flowchart outlining the study design and steps.

The study design and procedural steps are summarized in Figure 1 as a flowchart. The final study cohort consisted of 426 adult COVID-19 patients, with their demographic characteristics presented in Table 1. The cohort included 227 males (53.3%) and 199 females (46.7%), with no statistically significant difference in mean age between sexes (male: 54.98 ± 0.76 years; female: 55.18 ± 0.51 years; *p* = 0.7166). The overall median age was 56 years, ranging from 19 to 80 years. Mean BMI was significantly higher in females (31.49 ± 0.57) compared to males (28.88 ± 0.37; *p* < 0.001). Smoking habits varied by sex (*p* < 0.001); 91.96% of females and 75.74% of males reported never smoking. The rates of current or former smokers were low, with no significant differences in lab results.

**Figure 2:**
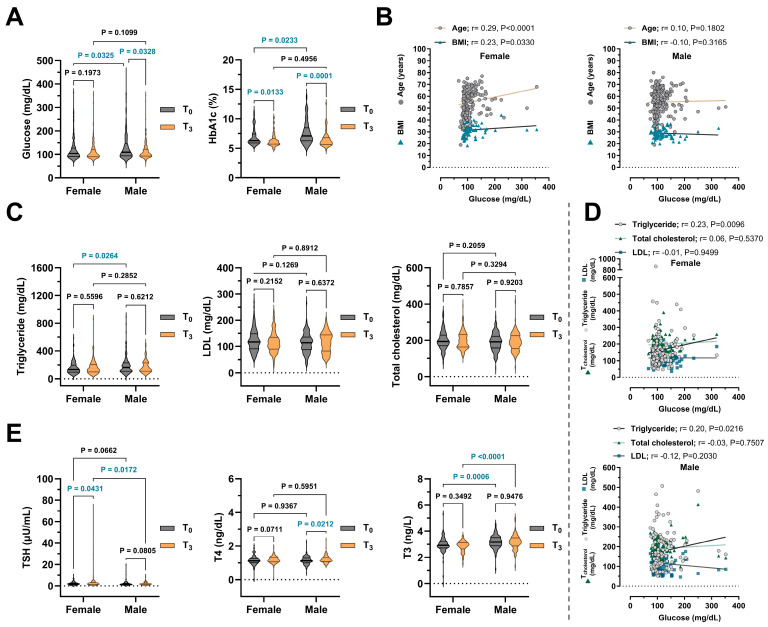
Longitudinal changes in metabolic and endocrine markers of COVID-19 patients. PCR-confirmed individuals categorized into two groups: female and male. Laboratory data were recorded at four intervals: baseline (T_0_), 18 months (T_1_), 24 months (T_2_), and 30 months (T_3_). (**A**) Glucose and Hemoglobin A1c (HbA1c) levels measured at T_0_ and T_3_ timepoints. (**B**) Spearman correlation analyses of glucose levels with age and body mass index (BMI). (**C**) Serum triglycerides, low-density lipoprotein (LDL) and total cholesterol levels measured at T_0_ and T_3_ timepoints. (**D**) Spearman correlation analyses of glucose levels with triglyceride, LDL, and total cholesterol levels. (**E**) Thyroid-stimulating hormone (TSH), thyroxine (T4), and triiodothyronine (T3) levels measured at T_0_ and T_3_ timepoints. The data are presented as mean ± SEM in violin plot graphs. Blue-colored *p* values denote statistically significant findings (*p* < 0.05).

Inter-sex comparisons were evaluated independently at each time interval. Accordingly, conclusions were drawn only from *p*-values calculated separately for T0, T1, T2, and T3, as pooled analyses may not accurately reflect sex-related differences. Over the 18-month follow-up (T0–T3), the cohort showed a significant reduction in fasting glucose levels, with values decreasing from 126.00 to 113.60 mg/dL (*p* = 0.017). Baseline glucose levels were higher in males than females (*p* = 0.033), though sex differences were not significant at later time points (Table 2). Glucose levels significantly declined in males (*p* = 0.033) but not in females (*p* = 0.197) (Figure 2A). Similarly, HbA1c levels decreased significantly from 7.26% to 6.43% in the total cohort (*p* < 0.001), with higher baseline values in males (*p* = 0.023). Both sexes exhibited significant reductions over time, though levels remained consistently higher in males (Figure 2A). Sex-stratified correlations showed that in females, glucose levels were positively associated with age (r = 0.29, *p* < 0.001) and BMI (r = 0.23, *p* = 0.033). No such associations were observed in males (Figure 2B). These results suggest improved glycemic control post-COVID-19 vaccination, particularly in males, and highlight age and BMI as important metabolic determinants in females.

In contrast to the marked changes in glucose metabolism, serum lipid parameters showed consistent, albeit non-statistically significant, changes over time. Triglyceride levels fluctuated across timepoints without significant within-group variation (*p* = 0.5726) (Table 3), though females had significantly lower baseline values compared to males (*p* = 0.0264) (Figure 2C). Neither total cholesterol nor LDL levels exhibited significant intra-group changes from T_0_ to T_3_ (*p* > 0.500); no sex differences were statistically significant at endpoint (Figure 2C). Correlation analyses (Figure 2D) showed a modest positive correlation between glucose and triglyceride levels in females (r= 0.23, *p* = 0.001) and males (r = 0.20, *p* = 0.021). No significant associations were found between glucose and total cholesterol or LDL for either gender, indicating a specific link between glucose metabolism and triglycerides. These figures provide a visual comparison of the baseline and long-term follow-up (T3) data. The full numerical results for T1 and T2 are provided in the tables and analyzed.

Thyroid function parameters showed mild, sex-specific changes over 18 months, mainly in TSH and free T3 levels, while free T4 remained stable. Females had a significant increase in TSH from baseline (2.18 ± 0.13 μU/mL) to T_3_ (3.26 ± 0.58 μU/mL) (*p* = 0.043), but no significant change was seen in males (*p* = 0.081) (Figure 2E). Mean free T3 levels remained relatively stable in the overall cohort (*p* = 0.507). At the T_0_ timepoint, free T3 levels were higher in males (3.18 ± 0.05 ng/L) than in females (2.95 ± 0.06 ng/L) (*p* = 0.0006) and remained elevated during the 30-month follow-up (*p* = 0.006 for T_1_, *p* < 0.0001 for T_2_ and T_3_) (Table 3). T4 levels increased slightly in the total population over time (*p* = 0.010), but not significantly between males and females (Table 3). Males showed a significant rise in T4 levels from T_0_ (1.15 ± 0.02 ng/dL) to T_3_ (1.21 ± 0.02 ng/dL) (*p* = 0.021) (Figure 2E).

**Figure 3:**
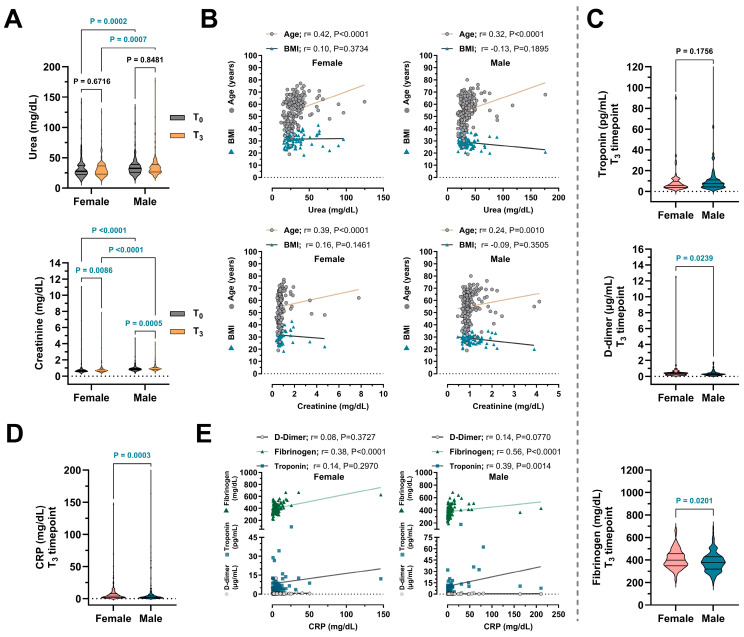
Longitudinal changes in inflammation, renal, and coagulation biomarkers of COVID-19 patients. PCR-confirmed individuals were categorized into two groups: female and male. Laboratory data were recorded at four intervals: baseline (T0), 18 months (T1), 24 months (T2), and 30 months (T3). (**A**) Serum urea and creatinine levels measured at T0 and T3 timepoints. (**B**) Spearman correlation analyses of urea and creatinine levels with age and body mass index (BMI). (**C**) Troponin, D-dimer, and fibrinogen levels measured at T3 timepoint. (**D**) C-reactive protein (CRP) levels at T3 timepoint. (**E**) Spearman correlation analyses of CRP levels with D-dimer, fibrinogen, and troponin levels. The data are presented as mean ± SEM in violin plot graphs. Blue-colored *p* values denote statistically significant findings (*p* < 0.05).

Renal function markers showed mild but significant sex-related differences over time. Urea levels remained stable in the overall cohort (*p* = 0.898) and within each sex (male: *p* = 0.848; female: *p* = 0.672), though females consistently exhibited lower levels than males at all time points (*p* < 0.01) (Figure 3A, Table 4). Serum creatinine levels increased significantly from T0 to T3 in both sexes (female: *p* = 0.009; male: *p* = 0.001), with males showing consistently higher values throughout (*p* < 0.001). These findings suggest subtle sex-based differences in renal adaptation post-infection. Correlation analysis revealed moderate positive associations between age and both urea and creatinine levels in males and females (*p* < 0.001), while BMI showed no significant correlation in either group (Figure 3B).

Cardiovascular and thrombo-inflammatory biomarkers demonstrated distinct sex-specific patterns (Figure 3C). D-dimer levels remained stable over time (*p* = 0.905), though females had significantly higher values than males at T3 (*p* = 0.024), suggesting delayed coagulative resolution. Fibrinogen levels declined overall (*p* < 0.001) but remained consistently higher in females across all timepoints (*p* < 0.05) (Table 4). Troponin levels decreased significantly in both sexes (female: *p* = 0.001; male: *p* = 0.003), yet males exhibited higher values during early follow-up, indicating greater subclinical cardiac stress, though this difference diminished by T3 (*p* = 0.176). Hypertension prevalence remained stable from baseline to T3 (*p* = 0.577), with slightly higher—but not statistically significant—rates in females at both timepoints (Table 4). No clear sex-specific trends were observed in hypertension post-COVID-19.

Systemic inflammation markers showed a significant decline in CRP levels over time (*p* = 0.001), with males exhibiting higher levels than females at T2 and T3 (*p* = 0.016 and *p* = 0.001, respectively) (Figure 3D, Table 5). At T3, CRP correlated moderately with fibrinogen in both sexes and with troponin in males, indicating a link between inflammation and vascular or myocardial stress during recovery (Figure 3E). Hematological parameters remained largely stable. WBC counts did not change significantly over time (*p* = 0.422), but males consistently had higher values, particularly at T1 (*p* = 0.001). Neutrophil percentages were comparable across sexes and timepoints (*p* = 0.194). A modest but significant increase in lymphocyte percentages was observed in females at T2 (*p* = 0.006), possibly reflecting transient sex-related differences in adaptive immunity post-COVID.

## 4. Discussion

This study presents a detailed longitudinal evaluation of metabolic, endocrine, renal, cardiovascular, and inflammatory parameters in COVID-19 survivors who had received at least two doses of vaccination. The analysis highlights sex-based differences in post-infection physiological adaptation over an 18-month follow-up period. Overall, several key biomarkers exhibited statistically and biologically relevant variations between male and female participants, underscoring the importance of considering sex as a biological variable in post-COVID-19 monitoring (see Table 2, Table 3, Table 4 and Table 5, Figure 2 and Figure 3).

One of the most consistent findings was a significant reduction in fasting glucose and HbA1c levels over time, which was more pronounced among male participants. Notably, several baseline parameters—including HbA1c, triglycerides, free T3, and urea—were higher in males compared to females (see Table 2, Table 3 and Table 4). These differences likely reflect a combination of innate sex-specific physiological variations, such as differences in body composition, hormonal milieu, and renal function, and potential acute effects of SARS-CoV-2 infection. Although the infection may have contributed to the initial increase in these biomarkers, the observed sex-specific baseline trends generally align with recognised biological differences between men and women. Positive correlations between glucose levels and both age and BMI, particularly in females, suggest a complex interplay between adiposity, hormonal environment, and metabolic regulation. Previous reports have highlighted that females are more prone to developing long COVID symptoms, including metabolic and autonomic disturbances [11], which may be attributed to sex-based immunometabolic differences [6]. Additionally, positive correlations were observed between glucose levels and both age and BMI in females, suggesting a more complex interplay between the hormonal milieu, adiposity, and metabolic regulation (see Table 2, Figure 2B).

Lipid parameters remained largely unchanged over time. Total cholesterol and LDL levels showed no significant intra-group variation. While triglycerides fluctuated slightly, male participants initially demonstrated higher levels than females, which gradually declined over time, whereas females showed a sustained increase by T3. These trends may indicate differential metabolic recovery or lingering inflammatory responses between sexes post-COVID-19, in line with known sex-specific metabolic trajectories. Although females are generally known to exhibit lower baseline triglyceride levels due to inherent sex-related differences in lipid metabolism [17], our findings suggest a dynamic and sex-specific trajectory of triglyceride responses following COVID-19. In line with previous observations reporting elevated triglyceride levels in the acute phase of infection [18,19], male participants in our cohort initially demonstrated significantly higher levels compared to females (see Table 3, Figure 2C). Interestingly, while triglyceride concentrations gradually declined in males over time, a sustained and progressive increase was noted in females by the T3 timepoint. This pattern may indicate differential metabolic recovery or lingering inflammatory responses between sexes in the post-acute phase of COVID-19. The observed positive correlation between glucose and triglycerides in both sexes may reflect shared metabolic pathways such as insulin resistance [20]. These findings align with prior reports indicating that lipid recovery following COVID-19 is often heterogeneous and tends to lag behind improvements in glucose metabolism, possibly due to persistent hepatic and inflammatory alterations [21].

Thyroid hormone profiles remained within reference ranges in both sexes but revealed subtle distinctions (see Table 3, Figure 2E). Females demonstrated a modest but marked increase in TSH over time, whereas TSH levels in males remained stable. This divergence may indicate differential modulation of the hypothalamic-pituitary-thyroid axis, possibly driven by immune-endocrine interactions. Supportively, Haitao et al. [10] and Maecker et al. [3] have highlighted sex-specific immune responses and their potential endocrine repercussions, particularly in viral convalescence. The consistently higher free T3 levels observed in males may reflect differences in deiodinase activity or peripheral hormone conversion [22]; however, further mechanistic studies are warranted.

In terms of renal function, serum urea levels remained stable during follow-up, but creatinine levels increased significantly in both sexes, consistently higher in males (see Table 4). These findings are consistent with known sex-based physiological differences, particularly in muscle mass and glomerular filtration dynamics, which influence baseline creatinine levels [23]. Notably, age correlated positively with creatinine levels in both groups, suggesting that age-related renal changes are a relevant factor in long-term follow-up [24].

Cardiovascular biomarkers also exhibited sex-specific trends. Troponin levels declined over time in both sexes; however, males consistently exhibited higher concentrations, especially during the early follow-up phases. This is consistent with reports that cardiac stress markers, such as troponin, are more prognostically relevant in men during and after COVID-19 [25]. D-dimer levels remained stable overall, though lower values were recorded in males at endpoint, indicating potentially faster coagulative recovery (see Table 4). Interestingly, fibrinogen levels were consistently higher in females, in line with prior evidence suggesting that women experience prolonged inflammatory responses [26]. Hypertension rates remained stable over time in both sexes, with a slightly higher but not statistically significant prevalence observed in females (see Table 5). This finding is consistent with previous reports indicating that COVID-19 infection and/or vaccination has a minimal long-term impact on blood pressure [27].

CRP levels also declined significantly in both sexes, with males consistently exhibiting higher mean concentrations (see Table 5). This observation aligns with evidence that systemic inflammation tends to resolve following vaccination, although the kinetics of inflammatory resolution may differ by sex [11,26]. Notably, positive correlations between CRP and fibrinogen, as well as troponin, in males further support the interconnected roles of systemic inflammation, vascular reactivity, and myocardial stress during the post-viral recovery phase [25]. Besides, hematological indices, including WBC and neutrophil percentages, remained relatively stable over time, except for a temporary increase in lymphocytes observed in females (see Table 5). This suggests stable innate immune dynamics, accompanied by some sex-specific adaptive responses. Supportively, Green et al. [2] have reported that females may exhibit stronger immune responses to vaccines and infections, which could partially explain these observations.

Overall, in this COVID-19 cohort, male participants exhibited significantly higher baseline levels of fasting glucose, HbA1c, renal markers (urea and creatinine), and cardiovascular biomarkers (troponin) (see Table 2, Table 3, Table 4 and Table 5), reflecting a more pronounced acute physiological burden findings that are consistent with prior reports of heightened immediate COVID-19 severity among men, potentially driven by hormonal differences, immune response variability, or differential ACE2 expression [1,6,25,26]. Conversely, although the present investigation did not directly assess long COVID symptomatology, findings suggest that females may be prone to long COVID syndrome (see Table 2, Table 3, Table 4 and Table 5, Figure 2 and Figure 3). Growing evidence suggests that women may experience a higher frequency of prolonged post-infection manifestations and increased vulnerability, which has been attributed to distinctive immune modulation, hormonal influences, or sustained inflammatory processes extending beyond the acute phase [9,28,29]. These sex-specific patterns underscore the imperative for tailored clinical strategies addressing both the acute and extended sequelae of COVID-19.

This study has several limitations. Its retrospective, single-center design may limit generalizability. Baseline laboratory data were not available for certain inflammatory and cardiac biomarkers (D-dimer, fibrinogen, high-sensitivity troponin, CRP, WBC, NEU, LY), and hypertension data were incomplete at intermediate time points (T1 and T2), which may have influenced the interpretation of longitudinal trends. Hormonal status, including sex hormones (e.g., estrogen, testosterone), and timing of infection were not recorded, which may act as confounding factors in sex-specific analyses. Proinflammatory cytokines (e.g., IFN-γ, IL-1β, IL-6) were not assessed, limiting mechanistic insight. Subgroup analyses by vaccine type (mRNA vs. inactivated) were not feasible due to limited sample sizes, and SARS-CoV-2-specific immune response data were unavailable. No participants had documented reinfection either before enrollment or during follow-up. Clinical outcomes, such as long COVID symptom incidence and quality-of-life measures, were not collected, limiting real-world applicability. Finally, the absence of functional outcome measures restricts broader contextualization.

Despite these limitations, the 30-month follow-up revealed sex-specific differences in metabolic, endocrine, renal, cardiovascular, and inflammatory markers among vaccinated COVID-19 survivors (see Table 2, Table 3, Table 4 and Table 5, Figure 2 and Figure 3): males showed earlier alterations in metabolic and cardiac parameters, whereas females exhibited more persistent changes in thyroid and inflammatory profiles. These results highlight the potential value of sex-tailored post-COVID monitoring, emphasizing early metabolic screening in men and prolonged endocrine-inflammatory follow-up in women. Future prospective studies incorporating hormonal data, functional outcomes, and comprehensive cytokine profiling are warranted to inform personalized recovery strategies.

## Figures and Tables

**Table 1:** Demographic data, vaccine doses, and vaccine type of COVID-19 patients.

		Total Population (n = 426)		Female (n = 199)		Male (n = 227)		*p* Value
Age	Min–Max (Median)	19–80 (56)		19–77 (57)		19–80 (55) 55.36 ± 0.69		0.7166
	Mean ± SEM	55.18 ± 0.51		54.98 ± 0.76				
BMI	Min–Max (Median)	18.37–51.02 (29.81)		18.37–51.02 (31.25)		19.92–39.79 (28.69) 28.88 ± 0.37		<0.0001 ***
	Mean ± SEM	30.09 ± 0.34		31.49 ± 0.57				
Smoking	Current/n (%)	25 (5.87)		8 (4.02)		17 (7.49)		<0.0001 ***
	Former/n (%)	46 (10.80)		8 (4.02)		38 (16.77)		
	Never/n (%)	355 (83.33)		183 (91.96)		172 (75.74)		
Vaccine doses	Vaccine type ^#^	n	Doses	n	Doses	n	Doses	
2 times	Inactivated	149	51	82	33	67	18	
	mRNA		247		131		116	
3 times	Inactivated	167	132	74	64	93	68	
	mRNA		369		158		211	
4 times	Inactivated	84	167	36	91	48	76	
	mRNA		169		53		116	
5 times	Inactivated	21	70	6	21	15	49	
	mRNA		35		9		26	
6 times	Inactivated	5	13	1	2	4	11	
	mRNA		17		4		13	

*** *p* < 0.001. ^#^ Inactivated = SINOVAC, mRNA = BNT162b2/Comirnaty.

**Table 2:** Laboratory results on glucose metabolism of COVID-19 patients categorized by biological sex.

		Total Population	Female	Male	
		Min–Max (Median)	Min–Max (Median)	Min–Max (Median)	*p* Value
		Mean ± SEM	Mean ± SEM	Mean ± SEM	
Glucose (mg/dL)	T0	66–444 (105.00)	66–363 (103.50)	67–444 (109.50)	0.0325 *
		126.00 ± 2.66	119.80 ± 3.40	131.30 ± 3.96	
	T1	70–504 (99.00)	72–357 (100.00)	70–504 (99.00)	0.4604
		120.20 ± 2.67	119.00 ± 3.81	121.40 ± 3.76	
	T2	66–540 (100.00)	66–369 (100.00)	73–540 (102.00)	0.2118
		117.80 ± 2.35	114.70 ± 2.94	120.70 ± 3.59	
	T3	64–355 (101.00)	67–355 (99.00)	64–352 (103.00)	0.1099
		113.60 ± 2.04	111.9 ± 2.94	115.20 ± 2.83	
*p* Value		0.0171 *	0.5191	0.0289 *	
HbA1c (%)	T0	5.10–13.70 (6.60)	5.40–10.20 (6.30)	5.10–13.70 (7.10)	0.0233 *
		7.26 ± 0.17	6.89 ± 0.20	7.63 ± 0.27	
	T1	5.00–12.10 (6.20)	5.20–12.10 (6.20)	5.00–11.70 (6.20)	0.9252
		6.74 ± 0.11	6.71 ± 0.16	6.77 ± 0.15	
	T2	4.80–13.70 (6.10)	4.80–10.30 (6.10)	4.90–13.70 (6.20)	0.1650
		6.58 ± 0.12	6.41 ± 0.13	6.77 ± 0.19	
	T3	4.70–12.60 (6.10)	4.90–10.30 (6.00)	4.70–12.60 (6.10)	0.4956
		6.43 ± 0.10	6.35 ± 0.13	6.51 ± 0.16	
*p* Value		<0.0001 ***	0.0381 *	0.0014 **	

* *p* < 0.05, ** *p* < 0.01, *** *p* < 0.001. *p*-values represent comparisons between male and female participants at each respective time point as well as across all time points combined.

**Table 3:** Laboratory results on lipid profile and thyroid function of COVID-19 patients categorized by biological sex.

		Total Population	Female	Male	
		Min–Max (Median)	Min–Max (Median)	Min–Max (Median)	*p* Value
		Mean ± SEM	Mean ± SEM	Mean ± SEM	
LDL (mg/dL)	T0	31–271 (1171.00)	51–271 (117.00)	31–265 (114.00)	0.1269
		118.30 ± 2.57	123.70 ± 3.89	113.30 ± 3.36	
	T1	34–269 (117.00)	34–264 (117.00)	39–269 (116.00)	0.9093
		118.60 ± 2.24	118.80 ± 3.31	118.40 ± 3.06	
	T2	28–275 (119.00)	28–275 (118.50)	32–268 (119.50)	0.8960
		118.70 ± 2.24	119.50 ± 3.38	118.00 ± 2.99	
	T3	25–234 (115.00)	25–234 (110.00)	46–203 (118.00)	0.8912
		115.80 ± 2.51	116.40 ± 3.57	115.10 ± 3.54	
*p* Value		0.8264	0.6261	0.7223	
Triglyceride (mg/dL)	T0	41–892 (143.00)	41–588 (135.00)	41–892 (150.00)	0.0264 *
		174.60 ± 6.83	156.20 ± 7.66	191.30 ± 10.86	
	T1	34–1263 (142.00)	34–525 (140.00)	42–1263 (152.00)	0.4293
		170.10 ± 6.28	160.70 ± 7.05	178.30 ± 10.00	
	T2	33–602 (138.00)	33–602 (129.00)	34–585 (143.00)	0.4579
		162.00 ± 5.20	159.00 ± 7.68	164.40 ± 7.09	
	T3	48–862 (142.00)	48–862 (141.00)	52–507 (141.00)	0.2852
		169.80 ± 6.26	165.60 ± 9.46	174.00 ± 8.22	
*p* Value		0.5726	0.9133	0.3010	
Total cholesterol (mg/dL)	T0	47–357 (193.00)	76–357 (193.00)	47–328 (191.50)	0.7179
		194.60 ± 3.13	200.10 ± 4.70	189.90 ± 4.16	
	T1	69–377 (195.50)	69–377 (197.50)	90–321 (192.00)	0.1149
		196.20 ± 2.52	199.30 ± 3.56	193.50 ± 3.53	
	T2	53–331 (192.00)	103–314 (195.50)	53–331 (191.00)	0.8209
		192.70 ± 2.39	195.50 ± 3.39	190.50 ± 3.35	
	T3	52–413 (193.00)	112–390 (194.50)	52–413 (191.00)	0.9736
		195.30 ± 3.10	199.40 ± 4.36	191.30 ± 4.39	
*p* Value		0.6734	0.8518	0.6984	
TSH (μU/mL)	T0	0.06–19.90 (1.67)	0.06–10.04 (1.85)	0.08–19.90 (1.51)	0.0662
		2.11 ± 0.12	2.18 ± 0.13	2.05 ± 0.18	
	T1	0.02–55.10 (1.79)	0.02–55.10 (1.91)	0.04–12.30 (1.72)	0.1448
		2.25 ± 0.16	2.46 ± 0.31	2.07 ± 0.12	
	T2	0.05–35.80 (1.91)	0.05–27.40 (2.04)	0.18–35.80 (1.61)	0.1006
		2.38 ± 0.15	2.48 ± 0.20	2.29 ± 0.22	
	T3	0.01–75.40 (1.89)	0.10–75.40 (2.12)	0.25–10.20 (1.77)	0.0172 *
		2.69 ± 0.30	3.26 ± 0.58	2.14 ± 0.13	
*p* Value		0.0575	0.1849	0.3372	
T3 (ng/L)	T0	0.02–5.30 (3.04)	0.02–5.30 (2.93)	1.11–4.52 (3.18)	0.0006 ***
		3.07 ± 0.04	2.95 ± 0.06	3.18 ± 0.05	
	T1	1.43–5.44 (3.08)	1.43–4.19 (2.98)	1.87–5.44 (3.12)	0.0061 **
		3.06 ± 0.03	2.97 ± 0.05	3.15 ± 0.04	
	T2	1.89–4.45 (3.10)	1.94–4.45 (3.01)	1.89–4.25 (3.23)	<0.0001 ***
		3.10 ± 0.03	2.98 ± 0.04	3.20 ± 0.04	
	T3	1.34–4.99 (3.05)	1.53–3.64 (2.94)	1.34–4.99 (3.18)	<0.0001 ***
		3.02 ± 0.04	2.85 ± 0.05	3.17 ± 0.06	
*p* Value		0.5073	0.4780	0.6687	
T4 (ng/dL)	T0	0.01–2.22 (1.13)	0.01–2.01 (1.13)	0.40–2.22 (1.13)	0.9367
		1.15 ± 0.02	1.15 ± 0.02	1.15 ± 0.02	
	T1	0.37–2.15 (1.17)	0.37–1.98 (1.16)	0.64–2.15 (1.17)	0.8525
		1.19 ± 0.01	1.20 ± 0.02	1.18 ± 0.01	
	T2	0.07–2.06 (1.17)	0.07–1.84 (1.18)	0.68–2.06 (1.16)	0.3274
		1.19 ± 0.01	1.21 ± 0.02	1.18 ± 0.01	
	T3	0.09–2.36 (1.17)	0.09–1.96 (1.16)	0.82–2.36 (1.18)	0.5951
		1.20 ± 0.02	1.19 ± 0.02	1.21 ± 0.02	
*p* Value		0.0104 *	0.0715	0.1365	

* *p* < 0.05, ** *p* < 0.01, *** *p* < 0.001. *p*-values represent comparisons between male and female participants at each respective time point as well as across all time points combined.

**Table 4:** Laboratory results on renal function, cardiac, and coagulation functions of COVID-19 patients categorized by biological sex.

		Total Population	Female	Male	
		Min–Max (Median)	Min–Max (Median)	Min–Max (Median)	*p* Value
		Mean ± SEM	Mean ± SEM	Mean ± SEM	
Urea (mg/dL)	T0	11.80–141.20 (30.25)	11.80–141.20 (27.60)	13.90–132.00 (32.40)	0.0002 ***
		33.75 ± 0.78	32.23 ± 1.21	35.09 ± 1.00	
	T1	11.70–114.50 (29.80)	11.70–114.50 (27.90)	15.10–103.80 (31.80)	<0.0001 ***
		32.66 ± 0.64	31.25 ± 1.05	33.93 ± 0.77	
	T2	11.70–163.60 (30.20)	11.70–163.60 (28.05)	17.30–136.40 (31.80)	<0.0001 ***
		33.55 ± 0.82	32.61 ± 1.38	34.38 ± 0.94	
	T3	12.00–176.00 (30.60)	12.00–124.60 (28.60)	18.10–176.00 (32.50)	0.0007 ***
		33.72 ± 0.84	31.80 ± 1.14	35.42 ± 1.21	
*p* Value		0.8980	0.9214	0.9814	
Creatinine (mg/dL)	T0	0.34–11.02 (0.76)	0.34–11.02 (0.65)	0.42–4.64 (0.87)	<0.0001 ***
		0.87 ± 0.03	0.81 ± 0.07	0.92 ± 0.03	
	T1	0.31–10.17 (0.79)	0.31–10.17 (0.68)	0.33–3.47 (0.89)	<0.0001 ***
		0.89 ± 0.03	0.83 ± 0.06	0.95 ± 0.02	
	T2	0.33–6.87 (0.80)	0.33–6.87 (0.68)	0.52–4.55 (0.90)	<0.0001 ***
		0.90 ± 0.03	0.82 ± 0.05	0.98 ± 0.03	
	T3	0.37–7.79 (0.83)	0.37–7.79 (0.69)	0.46–4.13 (0.91)	<0.0001 ***
		0.94 ± 0.03	0.84 ± 0.06	1.02 ± 0.03	
*p* Value		0.0017 **	0.0349 *	0.0031 **	
D-dimer (μg/mL)	T1	0.01–15.28 (0.29)	0.01–6.72 (0.31)	0.01–15.28 (0.27)	0.4619
		0.61 ± 0.06	0.55 ± 0.07	0.66 ± 0.10	
	T2	0.01–10.10 (0.30)	0.01–2.20 (0.31)	0.01–10.10 (0.31)	0.4281
		0.45 ± 0.04	0.41 ± 0.03	0.49 ± 0.07	
	T3	0.01–12.50 (0.30)	0.01–12.50 (0.36)	0.01–1.71 (0.32)	0.0239 *
		0.45 ± 0.05	0.48 ± 0.10	0.35 ± 0.02	
*p* Value		0.9050	0.5902	0.8610	
Fibrinogen (mg/dL)	T1	94–935 (424.50)	163–751 (450.00)	94–935 (406.00)	0.0114 *
		447.20 ± 6.65	457.20 ± 7.97	438.30 ± 10.34	
	T2	168–736 (397.00)	168–666 (406.00)	203–736 (382.50)	0.0008 **
		407.00 ± 5.12	422.20 ± 6.82	393.80 ± 7.40	
	T3	189–685 (383.00)	219–666 (398.00)	189–685 (378.50)	0.0201 *
		394.10 ± 5.46	407.40 ± 7.82	382.90 ± 7.51	
*p* Value		<0.0001 ***	<0.0001 ***	0.0010 **	
Troponin (pg/mL)	T1	0–1008.00 (4.30)	0–318.10 (3.50)	0–1008.00 (4.84)	0.0226 *
		12.17 ± 4.23	8.44 ± 2.88	15.23 ± 7.33	
	T2	0–261.00 (4.86)	1.40–75.67 (4.13)	0–261.00 (5.87)	0.0517
		11.38 ± 2.14	7.04 ± 1.17	14.85 ± 3.70	
	T3	1.00–175.30 (6.81)	1.00–89.82 (5.77)	1.40–175.30 (7.32)	0.1756
		10.84 ± 1.73	9.20 ± 1.70	12.30 ± 2.89	
*p* Value		<0.0001 ***	0.0002 ***	0.0033 **	
		Yes/n (%)	Yes/n (%)	Yes/n (%)	*p* Value
		No/n (%)	No/n (%)	No/n (%)	
Hyper-tension	T0	170 (39.91)	89 (44.72)	81 (35.68)	0.0603
		256 (60.09)	110 (55.28)	146 (64.32)	
	T3	179 (42.02)	93 (46.73)	86 (37.89)	0.0766
		247 (57.98)	106 (53.27)	141 (62.11)	
*p* Value		0.5773	0.3289	0.6971	

* *p* < 0.05, ** *p* < 0.01, *** *p* < 0.001. *p*-values represent comparisons between male and female participants at each respective time point as well as across all time points combined.

**Table 5:** Laboratory results on blood routine and inflammation profile of COVID-19 patients categorized by biological sex.

		Total Population	Female	Male	
		Min–Max (Median)	Min–Max (Median)	Min–Max (Median)	*p* Value
		Mean ± SEM	Mean ± SEM	Mean ± SEM	
CRP (mg/dL)	T1	0.02–309.80 (4.34)	0.02–151.40 (5.53)	0.27–309.80 (3.52)	0.0702
		13.51 ± 1.35	11.21 ± 1.31	15.53 ± 2.26	
	T2	0.27–276.70 (3.37)	0.27–117.90 (4.43)	0.28–276.70 (2.77)	0.0159 *
		8.42 ± 1.47	7.68 ± 0.96	9.07 ± 2.02	
	T3	0.21–210.60 (3.14)	0.21–146.60 (3.98)	0.23–210.60 (2.47)	0.0003 ***
		7.64 ± 0.88	7.32 ± 0.95	7.92 ± 1.42	
*p* Value		0.0003 ***	0.0435 *	0.0036 *	
WBC Count (×10^9^/L)	T1	0.88–24.10 (6.87)	1.76–20.13 (6.54)	0.88–24.10 (7.20)	0.0007 ***
		7.26 ± 0.12	6.82 ± 0.14	7.65 ± 0.18	
	T2	1.10–24.42 (6.87)	3.34–24.42 (6.72)	1.10–16.48 (7.16)	0.0025 **
		7.28 ± 0.11	7.04 ± 0.17	7.50 ± 0.14	
	T3	2.69–20.36 (7.02)	2.69–20.36 (6.98)	3.41–16.80 (7.09)	0.3913
		7.41 ± 0.11	7.31 ± 0.17	7.49 ± 0.14	
*p* Value		0.4225	0.0931	0.9035	
NEU Count (×10^3^/µL)	T1	5.87–93.40 (60.05)	5.87–89.70 (60.20)	36.30–93.40 (59.90)	0.9535
		60.42 ± 0.49	59.95 ± 0.72	60.84 ± 0.67	
	T2	5.49–91.20 (59.25)	25.30–91.20 (58.30)	5.49–85.10 (60.10)	0.1503
		59.33 ± 0.46	58.81 ± 0.64	59.78 ± 0.66	
	T3	19.90–90.40 (58.80)	19.90–90.40 (59.10)	40.10–84.00 (58.15)	0.5921
		59.25 ± 0.44	59.19 ± 0.67	59.32 ± 0.59	
*p* Value		0.1939	0.2267	0.2909	
LY Count (%)	T1	2.30–76.40 (29.70)	8.20–76.40 (30.20)	2.30–53.80 (29.40)	0.3054
		29.82 ± 0.43	30.56 ± 0.62	29.16 ± 0.60	
	T2	6.20–72.50 (30.80)	6.20–72.50 (32.07)	10.70–59.40 (30.02)	0.0064 **
		30.98 ± 0.41	32.07 ± 0.59	30.02 ± 0.56	
	T3	7.40–78.00 (31.30)	7.40–78.00 (31.30)	7.90–51.50 (31.25)	0.4812
		31.07 ± 0.42	31.72 ± 0.65	30.50 ± 0.55	
*p* Value		0.0692	0.0972	0.2235	

* *p* < 0.05, ** *p* < 0.01, *** *p* < 0.001. *p*-values represent comparisons between male and female participants at each respective time point as well as across all time points combined.

## Data Availability

The data that support the findings of this study are available from the corresponding author upon reasonable request.
